# Supplementary material for: Heparin-based hydrogel scaffolding alters the transcriptomic profile and increases the chemoresistance of MDA-MB-231 triple-negative breast cancer cells
Source: Biomater Sci. 2020 Feb 13;8(10):2786–96. doi: 10.1039/c9bm01481k (PMC7497406; doi:10.1039/c9bm01481k)
Supplement: Supplementary file 2 [file BM-008-C9BM01481K-s002.zip › Supplementary File 4/HP-BhydrogelvControl/Pathways/my_analysis.Gsea.1545201079907/HALLMARK_MYOGENESIS.html]

Details for gene set HALLMARK\_MYOGENESIS[GSEA]

|  || Dataset | expr.class.cls#HYD\_versus\_CONTROL.class.cls#HYD\_versus\_CONTROL\_repos |
| Phenotype | class.cls#HYD\_versus\_CONTROL\_repos |
| Upregulated in class | HYD |
| GeneSet | HALLMARK\_MYOGENESIS |
| Enrichment Score (ES) | 0.41245607 |
| Normalized Enrichment Score (NES) | 1.6364996 |
| Nominal p-value | 0.00125 |
| FDR q-value | 0.028340349 |
| FWER p-Value | 0.069 |
Table: GSEA Results Summary

  

Fig 1: Enrichment plot: HALLMARK\_MYOGENESIS      
 Profile of the Running ES Score & Positions of GeneSet Members on the Rank Ordered List

  

| PROBE | DESCRIPTION (from dataset) | GENE SYMBOL | GENE\_TITLE | RANK IN GENE LIST | RANK METRIC SCORE | RUNNING ES | CORE ENRICHMENT || 1 | COX7A1 | na |  |  | 9 | 4.195 | 0.0343 | Yes |
| 2 | DMPK | na |  |  | 217 | 2.888 | 0.0474 | Yes |
| 3 | STC2 | na |  |  | 313 | 2.647 | 0.0644 | Yes |
| 4 | COL1A1 | na |  |  | 499 | 2.347 | 0.0742 | Yes |
| 5 | PDE4DIP | na |  |  | 540 | 2.291 | 0.0911 | Yes |
| 6 | CHRNG | na |  |  | 656 | 2.157 | 0.1029 | Yes |
| 7 | PICK1 | na |  |  | 659 | 2.154 | 0.1207 | Yes |
| 8 | NAV2 | na |  |  | 906 | 1.942 | 0.1239 | Yes |
| 9 | NOTCH1 | na |  |  | 1056 | 1.835 | 0.1313 | Yes |
| 10 | PLXNB2 | na |  |  | 1060 | 1.833 | 0.1464 | Yes |
| 11 | TAGLN | na |  |  | 1147 | 1.771 | 0.1566 | Yes |
| 12 | AEBP1 | na |  |  | 1189 | 1.745 | 0.1689 | Yes |
| 13 | SPEG | na |  |  | 1211 | 1.733 | 0.1822 | Yes |
| 14 | KIFC3 | na |  |  | 1226 | 1.723 | 0.1957 | Yes |
| 15 | TPM2 | na |  |  | 1256 | 1.709 | 0.2084 | Yes |
| 16 | ENO3 | na |  |  | 1277 | 1.702 | 0.2214 | Yes |
| 17 | COL15A1 | na |  |  | 1473 | 1.593 | 0.2244 | Yes |
| 18 | COL4A2 | na |  |  | 1514 | 1.570 | 0.2354 | Yes |
| 19 | DAPK2 | na |  |  | 1682 | 1.497 | 0.2390 | Yes |
| 20 | TSC2 | na |  |  | 1754 | 1.468 | 0.2475 | Yes |
| 21 | AKT2 | na |  |  | 1769 | 1.459 | 0.2588 | Yes |
| 22 | ADCY9 | na |  |  | 1794 | 1.444 | 0.2696 | Yes |
| 23 | GADD45B | na |  |  | 1972 | 1.375 | 0.2717 | Yes |
| 24 | MAPK12 | na |  |  | 2008 | 1.357 | 0.2811 | Yes |
| 25 | CKMT2 | na |  |  | 2197 | 1.276 | 0.2818 | Yes |
| 26 | ITGA7 | na |  |  | 2333 | 1.230 | 0.2850 | Yes |
| 27 | MYH3 | na |  |  | 2421 | 1.202 | 0.2904 | Yes |
| 28 | SPTAN1 | na |  |  | 2525 | 1.164 | 0.2946 | Yes |
| 29 | BAG1 | na |  |  | 2573 | 1.152 | 0.3017 | Yes |
| 30 | TCAP | na |  |  | 2620 | 1.137 | 0.3087 | Yes |
| 31 | MYLK | na |  |  | 2690 | 1.120 | 0.3144 | Yes |
| 32 | FLII | na |  |  | 2740 | 1.106 | 0.3210 | Yes |
| 33 | SIRT2 | na |  |  | 2749 | 1.102 | 0.3297 | Yes |
| 34 | MYO1C | na |  |  | 2814 | 1.081 | 0.3353 | Yes |
| 35 | ATP2A1 | na |  |  | 2897 | 1.064 | 0.3399 | Yes |
| 36 | VIPR1 | na |  |  | 2917 | 1.059 | 0.3476 | Yes |
| 37 | BIN1 | na |  |  | 2965 | 1.045 | 0.3539 | Yes |
| 38 | SLC6A8 | na |  |  | 3116 | 1.006 | 0.3543 | Yes |
| 39 | LDB3 | na |  |  | 3145 | 1.002 | 0.3612 | Yes |
| 40 | ERBB3 | na |  |  | 3335 | 0.957 | 0.3592 | Yes |
| 41 | SMTN | na |  |  | 3348 | 0.954 | 0.3665 | Yes |
| 42 | ADAM12 | na |  |  | 3349 | 0.953 | 0.3744 | Yes |
| 43 | LSP1 | na |  |  | 3350 | 0.953 | 0.3823 | Yes |
| 44 | SPHK1 | na |  |  | 3452 | 0.927 | 0.3847 | Yes |
| 45 | LAMA2 | na |  |  | 3545 | 0.908 | 0.3874 | Yes |
| 46 | COL6A3 | na |  |  | 3618 | 0.889 | 0.3910 | Yes |
| 47 | PFKM | na |  |  | 3663 | 0.879 | 0.3960 | Yes |
| 48 | KLF5 | na |  |  | 3767 | 0.855 | 0.3977 | Yes |
| 49 | PSEN2 | na |  |  | 3877 | 0.838 | 0.3989 | Yes |
| 50 | PPFIA4 | na |  |  | 3914 | 0.830 | 0.4039 | Yes |
| 51 | SH2B1 | na |  |  | 4125 | 0.784 | 0.3994 | Yes |
| 52 | MB | na |  |  | 4181 | 0.774 | 0.4029 | Yes |
| 53 | CTF1 | na |  |  | 4321 | 0.748 | 0.4018 | Yes |
| 54 | PDLIM7 | na |  |  | 4395 | 0.733 | 0.4041 | Yes |
| 55 | ST5 | na |  |  | 4464 | 0.722 | 0.4065 | Yes |
| 56 | TEAD4 | na |  |  | 4558 | 0.700 | 0.4074 | Yes |
| 57 | MYH11 | na |  |  | 4639 | 0.687 | 0.4089 | Yes |
| 58 | ITGB5 | na |  |  | 4750 | 0.668 | 0.4087 | Yes |
| 59 | PYGM | na |  |  | 4784 | 0.662 | 0.4125 | Yes |
| 60 | AK1 | na |  |  | 4927 | 0.638 | 0.4103 | No |
| 61 | CAMK2B | na |  |  | 5007 | 0.624 | 0.4113 | No |
| 62 | HBEGF | na |  |  | 5567 | 0.542 | 0.3865 | No |
| 63 | LPIN1 | na |  |  | 5826 | 0.510 | 0.3772 | No |
| 64 | GNAO1 | na |  |  | 5870 | 0.504 | 0.3791 | No |
| 65 | HDAC5 | na |  |  | 5924 | 0.497 | 0.3805 | No |
| 66 | TPD52L1 | na |  |  | 6047 | 0.480 | 0.3781 | No |
| 67 | EPHB3 | na |  |  | 6614 | 0.403 | 0.3517 | No |
| 68 | SVIL | na |  |  | 7043 | 0.354 | 0.3322 | No |
| 69 | REEP1 | na |  |  | 7044 | 0.354 | 0.3351 | No |
| 70 | IFRD1 | na |  |  | 7053 | 0.353 | 0.3376 | No |
| 71 | SSPN | na |  |  | 7075 | 0.351 | 0.3395 | No |
| 72 | SORBS1 | na |  |  | 7693 | 0.284 | 0.3095 | No |
| 73 | TNNT1 | na |  |  | 7749 | 0.281 | 0.3089 | No |
| 74 | FST | na |  |  | 7803 | 0.272 | 0.3084 | No |
| 75 | PPP1R3C | na |  |  | 7987 | 0.249 | 0.3008 | No |
| 76 | CACNA1H | na |  |  | 8140 | 0.241 | 0.2949 | No |
| 77 | MEF2D | na |  |  | 8218 | 0.229 | 0.2927 | No |
| 78 | COL6A2 | na |  |  | 8304 | 0.219 | 0.2901 | No |
| 79 | CKB | na |  |  | 9267 | 0.121 | 0.2406 | No |
| 80 | SCHIP1 | na |  |  | 9768 | 0.069 | 0.2150 | No |
| 81 | TNNC2 | na |  |  | 9907 | 0.054 | 0.2082 | No |
| 82 | ABLIM1 | na |  |  | 9912 | 0.052 | 0.2084 | No |
| 83 | TGFB1 | na |  |  | 9937 | 0.048 | 0.2075 | No |
| 84 | MRAS | na |  |  | 10093 | 0.031 | 0.1997 | No |
| 85 | GAA | na |  |  | 10143 | 0.023 | 0.1973 | No |
| 86 | ITGB4 | na |  |  | 10164 | 0.020 | 0.1964 | No |
| 87 | FDPS | na |  |  | 10354 | 0.012 | 0.1866 | No |
| 88 | DTNA | na |  |  | 10478 | -0.002 | 0.1802 | No |
| 89 | FOXO4 | na |  |  | 10927 | -0.055 | 0.1571 | No |
| 90 | MYL6B | na |  |  | 11336 | -0.094 | 0.1365 | No |
| 91 | CRAT | na |  |  | 11449 | -0.107 | 0.1315 | No |
| 92 | CDKN1A | na |  |  | 11746 | -0.145 | 0.1172 | No |
| 93 | SORBS3 | na |  |  | 11842 | -0.156 | 0.1135 | No |
| 94 | SPDEF | na |  |  | 11987 | -0.171 | 0.1074 | No |
| 95 | PC | na |  |  | 12058 | -0.180 | 0.1052 | No |
| 96 | SOD3 | na |  |  | 12196 | -0.199 | 0.0996 | No |
| 97 | OCEL1 | na |  |  | 12284 | -0.211 | 0.0968 | No |
| 98 | CHRNB1 | na |  |  | 12825 | -0.261 | 0.0707 | No |
| 99 | TNNC1 | na |  |  | 13325 | -0.327 | 0.0472 | No |
| 100 | BHLHE40 | na |  |  | 13518 | -0.356 | 0.0401 | No |
| 101 | ACHE | na |  |  | 13587 | -0.367 | 0.0396 | No |
| 102 | EIF4A2 | na |  |  | 13623 | -0.374 | 0.0408 | No |
| 103 | PTP4A3 | na |  |  | 13686 | -0.384 | 0.0408 | No |
| 104 | ATP6AP1 | na |  |  | 13746 | -0.395 | 0.0410 | No |
| 105 | APP | na |  |  | 13765 | -0.397 | 0.0433 | No |
| 106 | CAV3 | na |  |  | 13926 | -0.415 | 0.0383 | No |
| 107 | SGCA | na |  |  | 13959 | -0.415 | 0.0401 | No |
| 108 | SYNGR2 | na |  |  | 14179 | -0.438 | 0.0323 | No |
| 109 | IGFBP7 | na |  |  | 14288 | -0.461 | 0.0304 | No |
| 110 | MAPRE3 | na |  |  | 14442 | -0.482 | 0.0264 | No |
| 111 | CLU | na |  |  | 14511 | -0.492 | 0.0269 | No |
| 112 | ACSL1 | na |  |  | 14604 | -0.507 | 0.0263 | No |
| 113 | GPX3 | na |  |  | 14790 | -0.536 | 0.0210 | No |
| 114 | NQO1 | na |  |  | 14968 | -0.557 | 0.0164 | No |
| 115 | SCD | na |  |  | 15219 | -0.600 | 0.0082 | No |
| 116 | DMD | na |  |  | 15243 | -0.605 | 0.0120 | No |
| 117 | GSN | na |  |  | 15576 | -0.665 | 0.0001 | No |
| 118 | TPM3 | na |  |  | 16003 | -0.746 | -0.0160 | No |
| 119 | RIT1 | na |  |  | 16098 | -0.769 | -0.0146 | No |
| 120 | FABP3 | na |  |  | 16170 | -0.782 | -0.0118 | No |
| 121 | IGFBP3 | na |  |  | 16184 | -0.784 | -0.0060 | No |
| 122 | RB1 | na |  |  | 16313 | -0.813 | -0.0060 | No |
| 123 | MEF2A | na |  |  | 16964 | -0.974 | -0.0320 | No |
| 124 | CNN3 | na |  |  | 17035 | -0.996 | -0.0274 | No |
| 125 | PKIA | na |  |  | 17099 | -1.014 | -0.0223 | No |
| 126 | MEF2C | na |  |  | 17207 | -1.054 | -0.0191 | No |
| 127 | SPARC | na |  |  | 17223 | -1.058 | -0.0111 | No |
| 128 | CFD | na |  |  | 17568 | -1.184 | -0.0194 | No |
| 129 | SH3BGR | na |  |  | 17933 | -1.341 | -0.0273 | No |
| 130 | PRNP | na |  |  | 18243 | -1.523 | -0.0309 | No |
| 131 | FHL1 | na |  |  | 18385 | -1.628 | -0.0248 | No |
| 132 | AGL | na |  |  | 18405 | -1.643 | -0.0122 | No |
| 133 | GABARAPL2 | na |  |  | 18501 | -1.706 | -0.0030 | No |
| 134 | WWTR1 | na |  |  | 18516 | -1.718 | 0.0105 | No |
| 135 | HSPB8 | na |  |  | 19137 | -3.038 | 0.0032 | No |
Table: GSEA details [plain text format]

  

Fig 2: HALLMARK\_MYOGENESIS      
 Blue-Pink O' Gram in the Space of the Analyzed GeneSet

  

Fig 3: HALLMARK\_MYOGENESIS: Random ES distribution      
 Gene set null distribution of ES for **HALLMARK\_MYOGENESIS**
